# Chronic Hepatitis E Virus Infection Without Liver Injury in a Patient with Chronic Kidney Disease

**DOI:** 10.3390/pathogens14050420

**Published:** 2025-04-26

**Authors:** Oliver Viera-Segura, Ilsy X. Duarte-López, Isidro Loera-Robles, Norberto Singh-Ríos, Arturo Calderón-Flores, Edgar D. Copado-Villagrana, Nora A. Fierro

**Affiliations:** 1 Instituto en Investigación en Ciencias Biomédicas, Centro Universitario de Ciencias de la Salud, Universidad de Guadalajara, Guadalajara 44340, Mexico; 2 Unidad de Medicina Familiar 5, Instituto Mexicano del Seguro Social, Nogales 84000, Mexico; 3 Departamento de Inmunología, Instituto de Investigaciones Biomédicas, Universidad Nacional Autónoma de México, Mexico City 04510, Mexico

**Keywords:** chronic hepatitis E virus infection, normal liver function, immunocompetent, Mexico

## Abstract

Hepatitis E virus (HEV), the causative agent of hepatitis E, is the leading cause of acute viral hepatitis worldwide; under immunosuppression, infection can lead to chronic liver disease. Furthermore, extrahepatic manifestations, particularly renal manifestations, are frequently associated with infection. This is important considering the global burden of chronic kidney disease (CKD). However, the study of chronic hepatitis E has been limited to liver disease, and its definition with respect to renal disease is still incomplete. Recently, through a protocol aimed at identifying HEV seroprevalence in a cohort of patients on hemodialysis, we incidentally identified HEV RNA in a patient with a history of alcoholism, diabetes mellitus, and essential systemic hypertension. In this study, we aimed to follow up this case to characterize hepatitis E in the context of CKD. Notably, we identified the development of chronic HEV genotype 3 infection without seroconversion or evidence of liver damage. Moreover, apparent immunocompetence was identified in the patient. Considering that HEV is still neglected in numerous countries and that it is not included in the differential diagnosis of kidney disease, our findings support the need to consider HEV infection in patients with renal disease, even in the absence of liver deterioration.

## 1. Introduction

Since the initial consideration of hepatitis E virus (HEV) as a causative agent of self-limiting infections and a predictor of poverty in the early 1980s, the understanding of this virus has changed significantly in recent years. This virus is currently recognized as a major issue in high-income regions and is the leading cause of acute hepatitis worldwide [1]. Moreover, HEV is a causative agent of chronic infections [2,3], and its capacity to affect multiple organs underscores the importance of studying it in detail, irrespective of its impact on liver function [4]. Projections estimate that 15 to 110 million individuals are experiencing ongoing hepatitis E and that approximately 939 million individuals in the global population have had the infection [5]. This information is derived from patients with liver disease, whereas estimates of adverse effects on extrahepatic HEV-associated organs are reported less frequently.

HEV is a single-stranded, positive-sense RNA virus of 7.2 kb that belongs to the *Hepeviridae* family. The main human-disease-causing strains belong to the *Paslahepevirus* genus. Among the *Paslahepevirus balayani* species, eight genotypes have been identified (HEV-1 to HEV-8), five of which affect humans [6]. The HEV-1 and HEV-2 genotypes cause outbreaks in low-income countries and are restricted to infecting humans [7]. The HEV-3 and HEV-4 genotypes are the leading causes of infections in industrialized countries, although HEV-3 circulates worldwide; these genotypes are associated with zoonotic transmission. HEV-7 is also zoonotic, and its circulation is limited to Asia [7,8]. In addition to enteric transmission, which is considered the most common source of HEV, zoonosis, vertical transmission during pregnancy, blood derivatives, and transplants are other potential transmission sources [2]. Notably, the progression of hepatitis E is influenced by the infectious genotype and the host immune response. In immunocompromised individuals, HEV-3 and HEV-4 infections can progress to chronic infections, as determined by the presence of the viral genome for more than three months [3]. These genotypes are also commonly related to the development of extrahepatic manifestations [3,9,10].

Chronic HEV infection may result in liver fibrosis and cirrhosis in immunosuppressed individuals [3]. In this respect, kidney transplant recipients are the patient group most commonly analyzed. In this group, the use of tacrolimus to prevent graft rejection is frequently related to the development of chronic HEV infection, and the minimum concentration of the drug is a determining factor in the development of this condition. Reducing the doses of immunosuppressive drugs is the first-line treatment for chronic HEV infection in post-transplant patients; however, in situations where this management is not feasible, the use of low doses of ribavirin represents an alternative to achieve viral elimination and avoid the consequences of the disease [11,12]. However, the use of ribavirin has limitations, which include relapse and the selection of ribavirin-insensitive viral variants, leading to treatment failure [13].

To date, information related to chronic HEV infection has been derived from its analysis in the context of liver function. In contrast, the understanding of this condition in extrahepatic organs is limited. No cases of chronic HEV infection have been identified in patients with chronic kidney disease (CKD) in the absence of immunosuppressive therapy, although there is evidence of isolated cases in which persistence of HEV RNA has been demonstrated in immunocompetent people [14,15,16,17,18]. Moreover, this condition in the context of normal liver function has not been reported.

CKD, a progressive disease characterized by changes in the structure and function of the kidneys, is a global public health problem, as denoted by its 9.1% global burden [19]. This is particularly important in low-income countries, where chronic conditions (i.e., diabetes) are increasingly common and directly impact renal deterioration [20]. In recent years, the group of hemodialysis (HD) patients has garnered interest in the study of hepatitis E; a greater seroprevalence has been demonstrated in these patients than in the general population [21,22,23]. Recently, we reported a seroprevalence of 14.9% for IgG anti-HEV antibodies in a cohort of HD patients [24].

Considering that renal manifestations are commonly found during hepatitis E and that this infection is still neglected in most of these countries, resulting in the lack of inclusion of HEV in the differential diagnosis of kidney disease, the study of HEV in the setting of CKD is necessary. Herein, we followed an incidentally identified HEV RNA-positive patient from a cohort of HD patients, with the aim of identifying elements involved in the characterization of hepatitis E in the kidney, particularly in CKD.

## 2. Case Description

The case in question involves a 45-year-old male who lives in the city of Nogales in northern Mexico. The patient presented with a history of alcoholism due to the consumption of more than 60 g of alcohol per day, on average, for 20 years, as well as diagnoses of type 2 diabetes mellitus 15 years prior, essential systemic hypertension 7 years prior, and chronic renal insufficiency 4 years prior. In addition to the CKD diagnosis, the patient was simultaneously evaluated for suspected liver failure associated with alcohol consumption; however, the diagnosis was ruled out on the basis of laboratory and office tests performed during the work-up.

**Figure 1:**
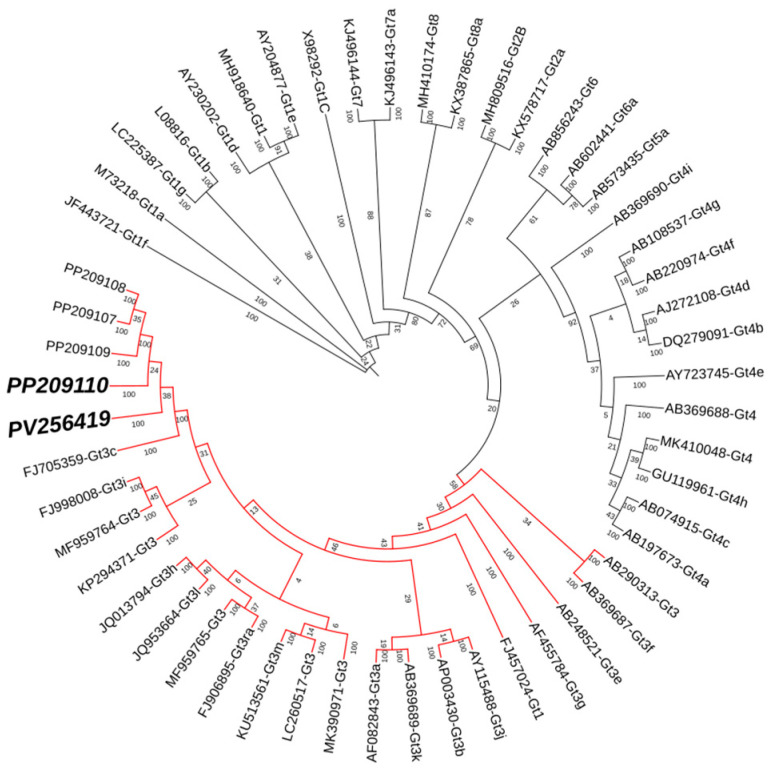
The phylogenetic analysis of ORF-1 HEV sequences collected over a one-year period. The tree was generated via Bayesian inference analysis. The sequence from the follow-up PV256419 is clustered together with the reference sequence genotype 3c (FJ705359-Gt3c); no sequence variation was found compared with the first sampling timepoint: PP209110.

Initially, the patient started renal replacement therapy with peritoneal dialysis, but after three months, he developed peritonitis, for which he had to seek treatment with HD. Two years later, a seroprevalence protocol for HEV was started among the patients in the HD unit; at that time, IgG and IgM anti-HEV antibodies were negative for this patient, but HEV RNA was detected (detection was based on the protocol from Wang et al. [25]). Partial HEV sequences via the Sanger method enabled phylogenetic analysis, classifying the virus as the HEV-3 genotype (GenBank accession number: PP209110). One year later, follow-up revealed that the viral RNA genome was still present; the amplification corresponded to the HEV ORF-1 (138 to 377 bp according to AF082843). The sequencing of a sample recovered during the follow-up allowed the identification of the HEV-3 genotype, which was sequenced and submitted to GenBank (accession number: PV256419) on the basis of the analysis conducted by aligning the reference sequences proposed by Smith et al. [26] via MAFFT v.7.525. The sequenced region corresponds to ORF-1, and phylogenetic analysis was carried out in MrBayes 3.2.7 for Bayesian inference. We used General Time Reversible with gamma-distribution rate to analyze the variation across sites and the proportion of invariable sites. We ran a simulation using 500,000 generations with Markov chain Monte Carlo. Seroconversion (IgM and IgG anti-HEV) was not detected during the follow-up. With this analysis, we provided evidence of the development of chronicity without seroconversion since the isolated viruses presented as 100% identical with the first isolation from the same patient (Figure 1).

A Bayesian inference revealed high posterior probabilities for branch support and convergence (ESS > 300). The sequences obtained in this study clustered together within the HEV-3 clade with 100% support, and the genetic distance between them was 0%, as determined by a pairwise distance matrix and comparison with reference sequences.

Importantly, the infection was incidentally found. Since the inclusion of the patient in the seroprevalence protocol for HEV, he has not manifested symptoms of hepatitis; abnormal liver function values have not been recognized, nor have morphological alterations been identified via ultrasound of the liver and bile ducts. Notably, according to Mexican guidelines, HD units routinely perform serology for the identification of human immunodeficiency virus (HIV), hepatitis B virus (HBV), and hepatitis C virus (HCV), but all these patient results were negative thus far. Table 1 shows the results of the comprehensive biochemical and laboratory evaluations performed after chronic HEV infection was identified. The normal values of aspartate aminotransferase (AST), alanine aminotransferase (ALT), and bilirubin stand out, as does the absence of fibrosis and hepatomegaly.

**Figure 2:**
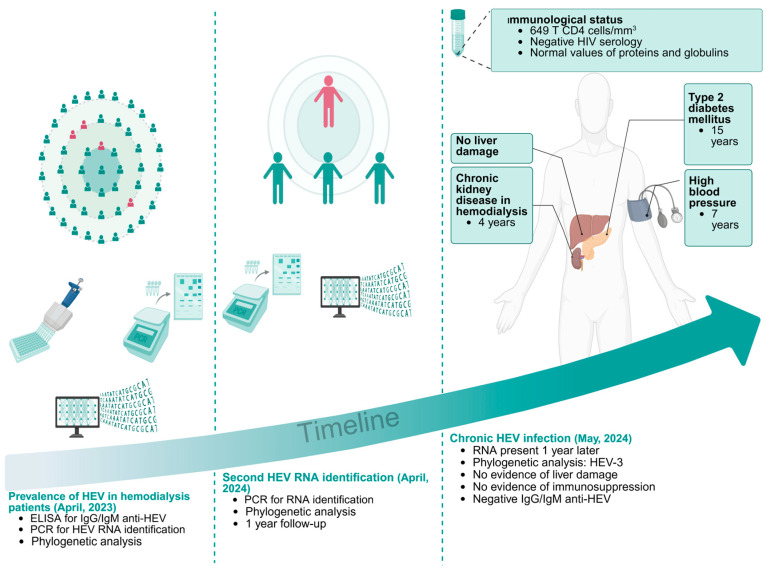
The timeline from the seroprevalence study to describing a chronic HEV infection case.

In terms of the patient’s immune status during follow-up, the CD4 T lymphocyte count was 649 cells per cubic millimeter, in addition to the normal values of proteins and total immunoglobulins. The above findings and the negative result of HIV serology suggested an immunocompetent status (Figure 2).

Despite not being diagnosed with anemia, the patient was not a candidate for ribavirin therapy because of deteriorating renal function and apparent immunocompetence, for which conservative treatment was preferred.

## 3. Discussion

Typically, HEV is considered as an exclusive cause of liver disease. Herein, we report an apparent immunocompetent patient with CKD and HEV chronic infection without evidence of liver manifestations.

Although chronic HEV infection has been associated mainly with immunosuppression, isolated cases in which the persistence of HEV RNA in immunocompetent individuals has been observed have been reported; concomitant conditions related to chronic liver damage have been identified in these reports [14,15,16,17,18]. Of particular interest for our study, Kanda et al. reported the case of a 70-year-old male with a history of alcohol consumption since the age of 12 who was admitted to Nihon Itabashi University Hospital in Tokyo. The patient was diagnosed with severe hepatitis caused by the HEV-3 genotype. This case is noteworthy because although the infection was resolved by day 34 and was not classified as chronic, the patient also had stage 4 CKD. Importantly, the patient was not considered a candidate for ribavirin treatment because of concerns about further reducing the glomerular filtration rate [27].

Herein, no evidence of immunocompromise was found. Undoubtedly, the lack of information regarding the patient’s immune status prior to the commencement of the study is a limitation. In that sense, it is important to consider that although patients with HD are not considered to be in overt immunosuppression, immune system dysfunction and inflammation are common in this group [28]. Moreover, diabetes and high consumption of alcohol might affect the immune response [29,30]. These characteristics may explain the seronegative results observed in the patient despite the presence of the viral genome and could be related to the development of chronic HEV infection.

Notably, viremia is infrequently reported in HD patients [22,23], and we detected HEV-3 infection in the serum for 12 months. With the results of the phylogenetic analysis, the possibility of reinfection can be ruled out, considering that the sequences in samples taken one year apart are practically identical, supported by a 0% p-distance and a convergence branch of 100% support. This genomic stability aligns with previous reports of HEV, which reported an estimated mutation rate of 1.5 base substitutions per site per year [31]. Notably, both patients’ sequences clustered with locally circulating HEV-3 strains previously reported by our group [32], supporting a community-acquired origin of infection. This finding is particularly significant, as these local strains were also identified in other hemodialysis (HD) patients from the same region [24], increasing the possibility of nosocomial transmission within dialysis units. Further epidemiological investigations are warranted to assess potential healthcare-associated exposure routes, such as contaminated medical equipment or blood products, given the potential immunocompromised status of this population. Our study is limited by single-patient observation and a lack of serial sampling to assess intrahost viral diversity. Future studies using next-generation sequencing (NGS) could elucidate minor variants and adaptation mechanisms during chronic HEV-3 infection in HD patients.

Interestingly, normal liver function was detected in the presence of HEV. This coincides with increasing evidence of neurological manifestations without direct involvement of the liver [4] and emphasizes the necessity of studying, in detail, the direct effect of infection on the kidney. Moreover, although the nature of this study does not allow us to associate the development of CKD with the virus, current evidence suggests that patients with chronic HEV infection are at risk of developing glomerulonephritis [33]. Evidence points toward the deposition of immune complexes of the HEV ORF 2 protein in the kidney as the cause rather than the viral replication in this tissue [33]. Therefore, large-scale, prospective studies are necessary to dissect the associations between CKD and HEV.

Our findings highlight the importance of incorporating HEV differential diagnosis in the setting of renal disease and revisiting the diagnosis of chronic HEV infection in the context of extrahepatic organs.

## Figures and Tables

**Table 1:** Laboratory and cabinet test results.

Parameter	Value (Reference)
Aspartate aminotransferase (AST)	24 UI/L (0–34)
Alanine aminotransferase (ALT)	19 UI/L (15–40)
Alkaline phosphatase	80 UI/L (35–129)
Direct bilirubin	0.1 mg/dL (0.0–0.2)
Indirect bilirubin	0.3 mg/dL (0.0–0.9)
Total bilirubin	0.4 mg/dL (0.3–1.2)
Creatinine	10.4 ↑mg/dL (0.5–1.2)
Glucose	88 mg/dL (74–106)
Uric acid	5.8 mg/dL (3.0–7.0)
Total cholesterol	127 ↓ mg/dL (140–220)
High-density lipoproteins	44 ↓ mg/dL (46–60)
Albumin	4.5 g/dL (3.5–5.2)
Calcium	12.4 ↑ mg/dL(8.3–10.6)
Magnesium	3.1 ↑ mg/dL (1.3–2.7)
Leukocytes	8.9 103/µL (4.5–12.0)
Hemoglobin	14.2 g/dL (11.5–18.0)
Hematocrit	43.8% (34.0–54.0)
Platelets	175 103/µL (150–450)
Lymphocytes	1.7 103/µL (0.6–3.4)
Neutrophils	6.1 103/µL (2.0–6.9)
CD4 T cells ^1^	649 cells/mm^3^ (500–1200)
Total proteins	7.8 g/dL (6.4–8.3)
Globulin	3.4 mg/dL (2.7–3.8)
HIV serology	Negative
HCV serology	Negative
HBV serology	Negative
Hepatomegaly ^2^	Negative
Liver fibrosis ^2^	Negative

^1^ Determined by flow cytometry, ^2^ determined by liver ultrasound.

## Data Availability

The sequences of the first and follow-up samples were submitted to GenBank under the accession numbers PP209110 and PV256419.
